# Circulating levels of sFlt-1 and AT1-AA in women with prior preeclampsia: a 10-year postpartum cross-sectional analysis (PERLA-Brazil study)

**DOI:** 10.1038/s41598-026-50409-1

**Published:** 2026-05-06

**Authors:** Thaíse Emilia Moreira da Silva, Isabella Macedo Costa, Ana Paula Silva Ferreira, Thays Santos da Costa, Luiza Oliveira Perucci, Juliano Armstrong Arnosti, Luci Maria Sant’Ana Dusse, Jussara Mayrink, Patrícia Nessralla Alpoim

**Affiliations:** 1 Department of Clinical and Toxicological Analysis, Faculty of Pharmacy, Federal University of Minas Gerais, Belo Horizonte, Brazil; 2 University of California, La Jolla, San Diego, CA USA; 3 Federal University of Paraná, Curitiba, Brazil; 4 Faculty of Medicine, Federal University of Minas Gerais, Belo Horizonte, Brazil; 5 Faculty of Pharmacy, University of Minas Gerais, St. Professor Moacir Gomes de Freitas, room 4156 – B3, Pampulha, Belo Horizonte, 31270-901 MG Brazil

**Keywords:** History of preeclampsia, SFlt-1, AT1-AA, Cardiovascular risk, Postpartum, Biomarkers, Cardiology, Diseases, Medical research

## Abstract

This study investigated whether soluble fms-like tyrosine kinase-1 (sFlt-1) and angiotensin II type 1 receptor autoantibodies (AT1-AA) – key mediators of preeclampsia (PE) pathophysiology – remain elevated ten years postpartum, thereby contributing to the long-term cardiovascular disease (CVD) risk in women with a history of PE. In a retrospective cohort nested within the PERLA-Brazil project, 205 women were enrolled: 103 with a documented history of PE (PH group) and 102 with normotensive pregnancies (NH group). Participants completed standardized interviews, underwent physical evaluations, and provided blood samples for sFlt-1 and AT1-AA quantification by enzyme-linked immunosorbent assay (ELISA), alongside comprehensive clinical and demographic data collection. No significant differences were observed in circulating sFlt-1 (90.25 ± 6.11 pg/mL in PH vs. 93.30 ± 5.54 pg/mL in NH, p = 0.886) or AT1-AA levels (6.80 ± 0.16 ng/mL in PH vs. 6.34 ± 0.18 ng/mL in NH, p = 0.060) between groups a decade after the index pregnancy. Clinical and biochemical data were collected and compared between groups, with multivariable models adjusted for age, body mass index, and educational attainment. As results, it was observed that women with severe PE history had higher systolic and diastolic blood pressure, higher low-density lipoprotein cholesterol, and a greater prevalence of hypertension. These findings suggest that the long-term cardiovascular burden after PE is not explained by increased plasma levels of sFlt-1 or AT1-AA, but may instead reflect durable downstream vascular and metabolic remodeling. The study underscores PE as a marker of future cardiovascular vulnerability and highlights the need to identify additional mechanisms and biomarkers underlying this risk.This highlights PE as an early-life stressor with persistent effects and underscores the need of lifelong CVD prevention strategies for affected women, as well as the importance of identifying additional biological or physiological markers associated with their sustained risk.

**Supplementary Information:**

The online version contains supplementary material available at 10.1038/s41598-026-50409-1.

## Introduction

Preeclampsia (PE) is a pregnancy-specific disorder that manifests after 20 weeks of gestation. It is characterized by new-onset hypertension accompanied by one or more of the following clinical features: proteinuria, thrombocytopenia, impaired liver or kidney function, pulmonary edema, new-onset headache not accounted for by alternative diagnoses and unresponsive to medication, or visual disturbances. PE affects 2 to 8% of pregnancies globally and is the major cause of maternal and infant mortality^1^.

Beyond its immediate perinatal risks, PE is considered a critical indicator for long-term maternal health. Women with a history of PE face a substantially increased risk of developing cardiovascular disease (CVD) later in life compared with those who experienced normotensive pregnancies^1–3^. This elevated risk encompasses a range of conditions, including chronic hypertension, ischemic heart disease, stroke, and venous thromboembolism, and persists throughout life after the affected pregnancy^1,4^. The mechanisms underlying this sustained cardiovascular vulnerability remain under intensive investigation, with growing evidence suggesting that PE serves as a “stress test” on the maternal cardiovascular system, revealing or even triggering an underlying predisposition to chronic diseases^1,3^.

From a pathophysiological standpoint, the development of PE is thought to involve an imbalance between angiogenic and antiangiogenic factors, along with activation of the renin-angiotensin system. A key antiangiogenic protein, soluble fms-like tyrosine kinase-1 (sFlt-1), is markedly elevated in the maternal circulation of women with PE^2,5–9^. This excess sFlt-1, primarily released from the ischemic placenta, binds to and neutralizes proangiogenic factors, such as vascular endothelial growth factor (VEGF) and placental growth factor (PlGF), leading to widespread endothelial dysfunction, hypertension, and proteinuria, which are hallmark features of the disorder^5–7,9^.

Agonistic autoantibodies against the angiotensin II type 1 receptor (AT1-AA) have been identified in women with PE^2,5,10,11^. These autoantibodies can activate AT1 receptors and have been implicated in the stimulation of sFlt-1 production^5,10,12^. Together, sFlt-1 and AT1-AA play central roles in the pathogenesis of PE and are also associated with its systemic manifestations, including hypertension, as well as with the development and progression of other cardiovascular pathologies^5,10,12^.

Notably, these biomarkers have been found to persist beyond pregnancy. Although sFlt-1 levels decline after delivery, studies indicate that they can remain significantly elevated in women with a history of PE, particularly in those with persistent AT1-AA, even up to one year postpartum^2,10^. Similarly, AT1-AA has been detected in a substantial proportion of women (17.2%) with prior PE more than a year after childbirth, in contrast to its absence in women who experienced uncomplicated pregnancies^2^. This sustained presence of AT1-AA and elevated sFlt-1 levels postpartum is clinically significant, as it may underlie the heightened angiotensin II sensitivity and endothelial dysfunction observed in these women, thereby promoting the development of chronic hypertension and other adverse cardiovascular outcomes later in life^1,2,6,10,11^.

Given the established link between PE and long-term cardiovascular disease, and the documented postpartum persistence of sFlt-1 and AT1-AA, a critical knowledge gap remains regarding their long-term trajectories. The current study represents a novel investigation, being the first to determine circulating levels of sFlt-1 and AT1-AA about a decade after an index preeclamptic pregnancy. This extended follow-up period of approximately ten years postpartum constitutes a unique and crucial contribution to understanding the prolonged biological footprint of PE and its implications for enduring cardiovascular vulnerability.

## Methods

### Study design and participants

This retrospective cohort study was conducted as part of the *Preeclampsia and Latent Cardiovascular Risk After Delivery (PERLA-Brazil)* project. A total of 205 women residing in Belo Horizonte, Minas Gerais, Brazil, were enrolled. Participants were categorized into two groups: 103 women with a documented history of PE (PH group) and 102 women with a history of normotensive pregnancies (NH group).

### Definition of preeclampsia and normotensive pregnancy history

Participants were rigorously categorized based on their obstetric history. Women in the PH group were defined as having experienced PE with severe features, according to the diagnostic criteria outlined in The American College of Obstetricians and Gynecologists (ACOG) Practice Bulletin No. 222^1^. This definition included new-onset hypertension (systolic blood pressure ≥ 160 mmHg and/or diastolic blood pressure ≥ 110 mmHg, measured at rest at least four hours apart) occurring after 20 weeks of gestation and up to 12 weeks postpartum, accompanied by one or more of the following features: proteinuria, impaired liver function, thrombocytopenia, new-onset headache unresponsive to medication and not attributable to alternative diagnoses, visual disturbances, renal dysfunction, or pulmonary edema. The (NH) group comprised women with a documented history of normotensive pregnancies, defined as blood pressure ≤ 120/80 mmHg, with no evidence of PE, gestational hypertension, chronic hypertension, or other high-risk prenatal conditions.

### Recruitment strategy and sample size determination

The recruitment process for this study spanned three distinct phases, beginning with outreach to 1,641 women. Of these, 1,082 met the predefined eligibility criteria, resulting in the enrollment of 205 individuals who provided consent and completed all required clinical evaluations at the Faculty of Pharmacy (FAFAR), Federal University of Minas Gerais (UFMG).

The initial recruitment phase targeted women who had previously participated in a Preeclampsia Research Group study at FAFAR/UFMG between 2008 and 2011^13,14^. Of 229 women contacted, only 10 attended the clinical assessments, which included comprehensive medical examinations and structured interviews. Due to the low response rate, the recruitment strategy was subsequently expanded.

Phase Two involved a meticulous review of 327 medical records from women diagnosed with PE during the same period at Júlia Kubitschek Hospital in Belo Horizonte, Brazil, following approval by the ethics committee (CAAE: 46792921.3.3002.5119). After applying strict inclusion and exclusion criteria, 226 women were deemed eligible; however, only six women consented to participate and completed the clinical evaluations following persistent telephone outreach.

The final phase implemented a broader outreach campaign using social media platforms, radio broadcasts, university-wide email communications, and strategically placed posters in high-traffic areas. This approach generated interest from 867 women, of whom 471 met the inclusion criteria. Ultimately, 127 women from this phase attended clinical evaluations and interviews at FAFAR/UFMG.

**Fig. 1:**
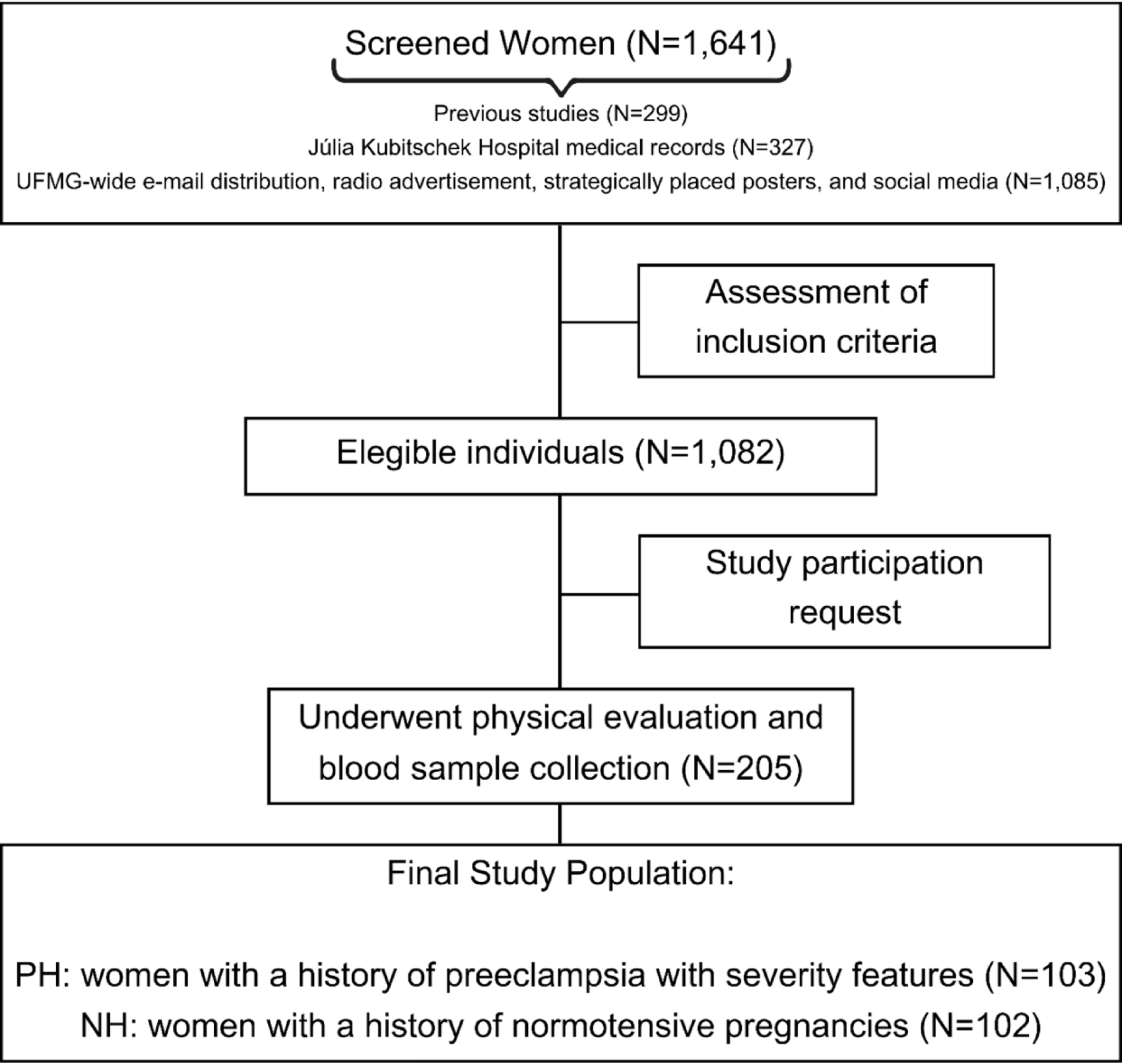
Participant recruitment and enrollment flowchart for the PERLA-Brazil study.

Overall, the final cohort predominantly consisted of self-referred volunteers (92%), with the remaining 8% recruited through active outreach efforts, as detailed in Fig. 1.

This flowchart illustrates the comprehensive participant recruitment and enrollment strategy employed for the PERLA-Brazil study, which ultimately resulted in a final cohort of 205 women.

The study was initially powered for low-density lipoprotein cholesterol (LDL-C) outcomes since LDL-C is a well-established marker of cardiovascular risk, as recognized by both the American Heart Association and the Brazilian Society of Cardiology (39, 40). Based on these parameters, at least 158 participants were needed, with 79 individuals allocated to each group. Post-hoc power analysis showed that, with *n* = 205, the study had 7.9% power to detect the observed difference in sFlt-1 (Cohen’s d = 0.053) and 43.5% power for AT1-AA (Cohen’s d = 0.397), both at α = 0.05 (two-tailed). The very low power for sFlt-1 is consistent with the essentially null effect size, indicating that a larger sample would be justified only if a meaningfully larger effect were expected.

### Inclusion and exclusion criteria

Inclusion criteria: Women were eligible if their pregnancy occurred between January 1 st, 2008, and December 31 st, 2017, and they were at least 18 years of age at the time of that pregnancy.

Exclusion criteria: Women were excluded if they had a history of chronic hypertension, diabetes, coagulation disorders, cancer, or autoimmune diseases during the index pregnancy. Furthermore, participants lacking a prenatal card and/or discharge summary were excluded, as these documents were essential for comprehensive verification of clinical data.

Regarding the diagnostic criteria employed for PE, we clarify that a retrospective reclassification of the subjects was not required. The main evolution in the diagnostic framework culminating in ACOG Practice Bulletin No. 222^1^ was the expansion of the definition of PE, most notably through the removal of mandatory proteinuria as an absolute diagnostic requirement. Because the 2020 guidelines broadened the diagnostic scope to include non-proteinuric presentations with systemic involvement, the historical diagnoses established during the study period (2008–2017), under the more restrictive criteria in force at the time, remain compatible with the updated framework. Moreover, clinical indicators of organ dysfunction, such as thrombocytopenia and impaired liver function, had already been established as severe features in earlier guidelines, thereby preserving the longitudinal consistency of the study sample with respect to disease severity.

### Data collection

Participant recruitment and data acquisition were conducted between February 2022 and August 2023. All participants provided written informed consent prior to enrollment. Data were obtained through a standardized interview, comprehensive physical evaluation, and blood sample collection conducted by trained research personnel at the Faculty of Pharmacy, Federal University of Minas Gerais (UFMG), located in Belo Horizonte, Brazil.

The research team meticulously gathered extensive information, including demographic data, family medical history, current health status, detailed obstetrical history, current medication usage, and lifestyle behaviors. To ensure the accuracy and reliability, self-reported information was verified against medical records, biochemical test results obtained from the pregnancy period, and prenatal/childbirth follow-up records provided by each participant.

The physical evaluation included the measurement of several key parameters: blood pressure (mmHg), body weight (kilograms), height (centimeters), waist and hip circumferences (centimeters), and body fat percentage. Blood pressure was measured using a Smartpro Q-400 TechLine^®^ automated device, with participants seated and at rest for at least 10 min prior to assessment. The presence of chronic conditions such as hypertension, diabetes mellitus, and hypercholesterolemia at the time of the study was identified primarily through self-reported continuous use of prescribed medications for these conditions.

### Laboratory analyses

Peripheral blood samples were collected from all participants in a non-fasting state using vacuum tubes with and without heparin as an anticoagulant. Following collection, samples were processed within two hours. Serum and plasma were separated by centrifugation at 3,000 x g for 10 min using a Heraeus Multifuge X1R (ThermoFisher Scientific^®^) centrifuge. The resulting samples were then aliquoted and immediately stored at −80 °C until analysis.

The serum lipid profile was measured using enzymatic colorimetric assays with Bioclin^®^ kits (total cholesterol: K083; HDL: K071; LDL: K088; Bioclin, Belo Horizonte, Brazil). Plasma sFlt-1 and AA-AT1 levels were quantified by enzyme-linked immunosorbent assay (ELISA) using commercial kits (sFlt-1: MBS3800911; AA-AT1: MBS722452; MyBioSource, San Diego, USA).

According to the manufacturer’s specifications (MyBioSource), the ELISA kit used to quantify sFlt1 levels in this study has a reported sensitivity (lower limit of detection) of 1 pg/mL. The intra-assay and inter-assay coefficients of variation (CVs) are both < 15%, indicating acceptable reproducibility and precision within and between assay runs. All measured maternal sFlt-1 concentrations in our study were above the assay’s lower limit of detection, ensuring that the reported values fall within the reliable quantification range of the assay. Two samples yielded values outside the assay’s dynamic range and were therefore excluded from downstream analyses to maintain data integrity.

### Covariate definitions

1. Hypertension: Defined based as current use of antihypertensive medication, or a documented systolic blood pressure ≥ 140 mmHg and/or diastolic blood pressure ≥ 90 mmHg, measured at least 15 min apart at rest during the study’s physical evaluation.
2. Body Mass Index (BMI): Categorized according to the World Health Organization (WHO) guidelines^15^ as:
  Normal weight: 18.50–24.99 kg/m².
  Overweight: 25.00–29.99 kg/m².
  Obese: ≥ 30.00 kg/m².
3. Medication Use: Continuous medication use for specific health conditions was identified through self-report. The following drugs were commonly reported:
  Diabetes Mellitus (DM): metformin, nebivolol, dapagliflozin.
  Hypercholesterolemia: rosuvastatin, atorvastatin, ezetimibe, orlistat, glucomannan, pitavastatin, simvastatin.
  Hypertension: enalapril, hydrochlorothiazide, amlodipine, losartan, captopril, spironolactone, indapamide, propranolol, atenolol, candesartan, methyldopa.
  Contraceptives: dehydroepiandrosterone, desogestrel, drospirenone, dienogest, ethinyl estradiol, levonorgestrel, medroxyprogesterone, chlormadinone, nomegestrol, cyproterone, gestodene, norethisterone, norelgestromin, etonogestrel.
4. Educational levels refer to the highest degree completed (Complete Elementary Education, Complete High School Education, Complete Higher Education), as self-reported.

### Statistical analysis

All statistical analyses were performed using IBM SPSS Statistics version 26.0 (IBM Corp., Armonk, NY, USA). The significance level was set at α = 0.05 (two-tailed) for all tests. The Central Limit Theorem indicates that the sampling distribution of the mean approaches normality as the sample size increases, even when the original variables are not normally distributed. In this context, the large sample (*n* = 205) provides sufficient statistical robustness to support the use of parametric methods for inferential analyses. Therefore, continuous variables were summarized as mean ± standard deviation (SD), while categorical variables were expressed as frequencies and percentages.

#### Descriptive statistics and group comparisons

Baseline characteristics of the study population were compared between the PH and NH groups using Student’s t-test for continuous variables, and Pearson’s chi-square test (or Fisher’s exact test when expected cell frequencies were < 5) for categorical variables. These comparisons assessed the demographic, anthropometric, clinical, laboratory, and medication-related comparability of the two groups.

#### Multivariate general linear model

The data were analyzed under the framework of the General Linear Models (GLM), with SBP, DBP, and LDL-C entered as dependent variables and pregnancy history (NH vs. PH group) as the fixed between-subjects factor. The first model included a broader set of covariates. To evaluate the robustness of pregnancy history effect under different adjustment strategies, the final model was intentionally specified as a more parsimonious model, retaining only the covariates considered most relevant to the outcomes under study. This reduced specification allowed us to examine whether the associations observed in the fully adjusted model were dependent on the inclusion of additional clinical and biochemical factors, or whether they remained stable under a simpler adjustment structure. In this context, the exclusion of some variables was motivated by model parsimony and sensitivity analysis rather than by statistical non-significance alone.

The analysis was performed in IBM SPSS Statistics using type III sums of squares, with predicted values saved for each outcome. Assumptions for multivariate analysis were assessed using Box’s M test for homogeneity of covariance matrices and Levene’s test for homogeneity of variances. Because the covariance matrices were heterogeneous and variance homogeneity was violated for DBP, multivariate inference was interpreted primarily using Pillai’s Trace, which is comparatively robust to assumption violations. Significant omnibus effects were followed by univariate tests of between-subjects effects for each outcome. Adjusted group differences were estimated using marginal means at the sample means of the covariates, and pairwise comparisons were Bonferroni-adjusted. Effect sizes are reported as partial eta squared.

## Results

A total of 205 women participated in this study: 103 women with a documented history of PE with severe features (PH group) and 102 women with a history of normotensive pregnancies (NH group). In the comparison between women with a history of normotensive pregnancy and those with a history of PE with severe features, no significant differences were observed for age (*p* = 0.230), number of pregnancies (*p* = 0.309), time since the index pregnancy (*p* = 0.155), overall medication use (*p* = 0.121), diabetes mellitus (*p* = 0.651), hypercholesterolemia (*p* = 0.306), contraceptive use (*p* = 0.815), HDL-C (*p* = 0.287), total cholesterol (*p* = 0.225), sFlt-1 (*p* = 0.886), or AT1-AA (*p* = 0.060). Significant differences between-group were found for educational attainment (*p* = 0.007), BMI (*p* = 0.031), hypertension (*p* < 0.001), SBP (*p* < 0.001), DBP (*p* < 0.001), and LDL-C (*p* = 0.001). The detailed clinical and laboratory characteristics of the study population are presented in Table 1.

**Table 1:** Characteristics of the study population.

Variable	NH	PH	*p*-value
Age (years)^†^	40.66 ± 5.99	39.67 ± 5.75	0.230
Educational attainment, n (%)^‡^			0.007*
Complete Elementary Education	2 (2%)	9 (9%)	
Complete High School Education	17 (17%)	29 (28%)	
Complete Higher Education	83 (81%)	64 (63%)	
BMI^‡^	26 ± 5	28 ± 5	0.031*
Normal weight, n (%) (18.50–24.99 kg/m²)	48 (47%)	32 (31%)	
Overweight, n (%) (25.00–29.99 kg/m²)	35 (15%)	40 (39%)	
Obese, n (%) (≥ 30.00 kg/m²)	18 (18%)	31 (30%)	
Overall medication use, n (%)^‡^	51 (50%)	62 (61%)	0.121
Diabetes mellitus	3 (3%)	2 (2%)	0.651
Hypercholesterolemia	3 (3%)	6 (6%)	0.306
Hypertension	5 (5%)	28 (27%)	< 0.001*
Contraceptive	14 (14%)	13 (13%)	0.815
Number of pregnancies^†^	2 ± 1	2 ± 1	0.309
Time since the index pregnancy (years)^†^	10 ± 3	10 ± 3	0.155
SBP (mmHg)^†^	123 ± 14	133 ± 20	< 0.001*
DBP (mmHg)^†^	81 ± 11	89 ± 15	< 0.001*
LDL-C (mg/dL)^†^	90 ± 33	107 ± 36	0.001*
HDL-C (mg/dL)^†^	67 ± 17	69 ± 21	0.287
Total cholesterol (mg/dL)^†^	180 ± 50	172 ± 36	0.225
sFlt-1 (pg/mL)^†^	92 ± 34	90 ± 41	0.886
AT1-AA (ng/mL)^†^	6 ± 1	7 ± 1	0.060

Data presented as †Mean ± SD, or ‡Frequency n (%). AT1-AA: angiotensin II type 1 receptor autoantibodies. NH: women with normotensive pregnancy history (*n* = 102, 49.7%). PH: women with preeclampsia with severe features history (*n* = 103, 50.3%). BMI: body mass index. SBP: systolic blood pressure. DBP: diastolic blood pressure. LDL-C: low-density lipoprotein cholesterol. HDLc: high-density lipoprotein cholesterol. sFlt-1: soluble fms-like tyrosine kinase-1 *p-value < 0.05 indicates statistical significance.

**Fig. 2:**
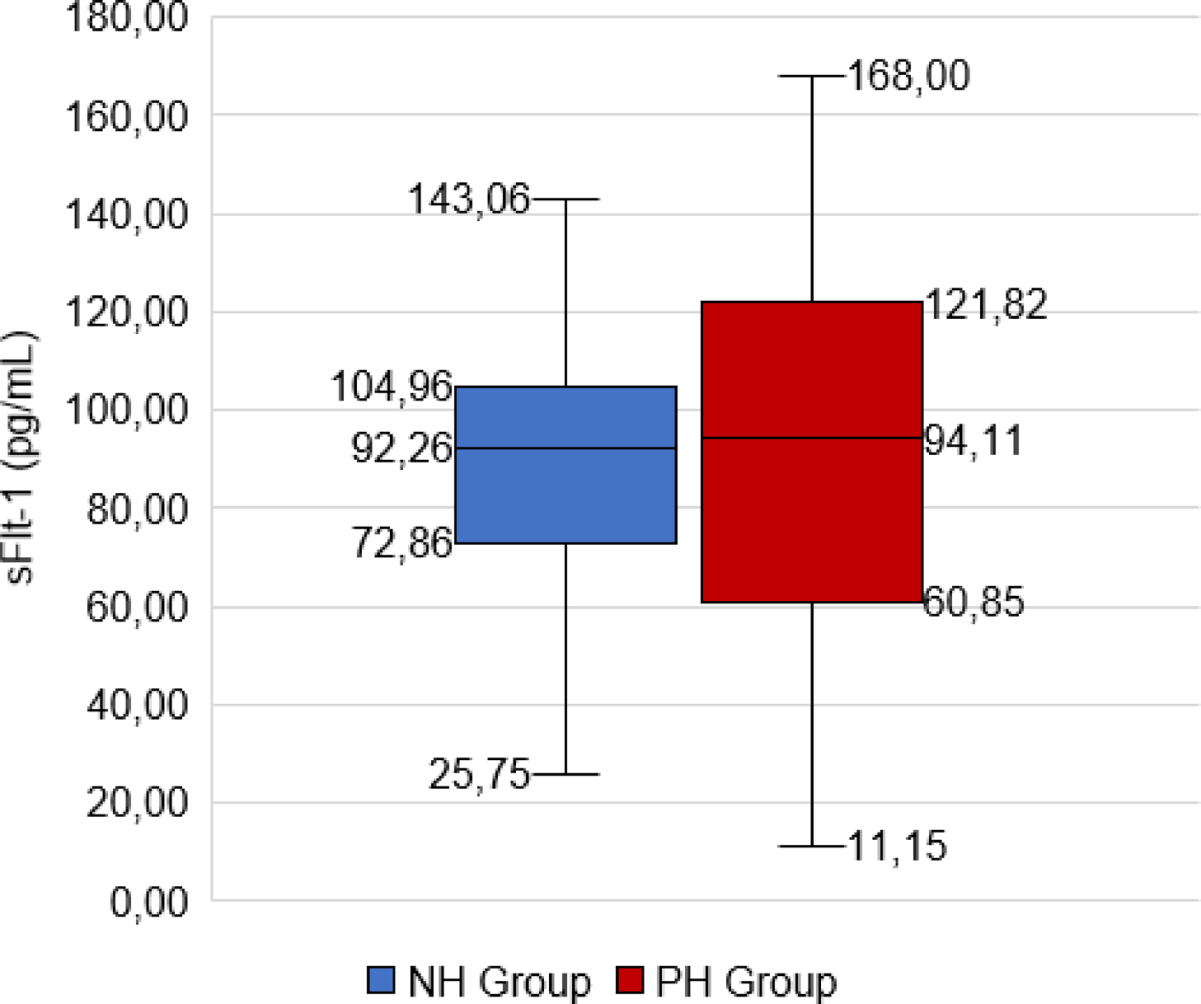
Circulating levels of soluble Fms-Like Tyrosine Kinase-1 (sFlt-1) a decade after the index pregnancy.

**Fig. 3:**
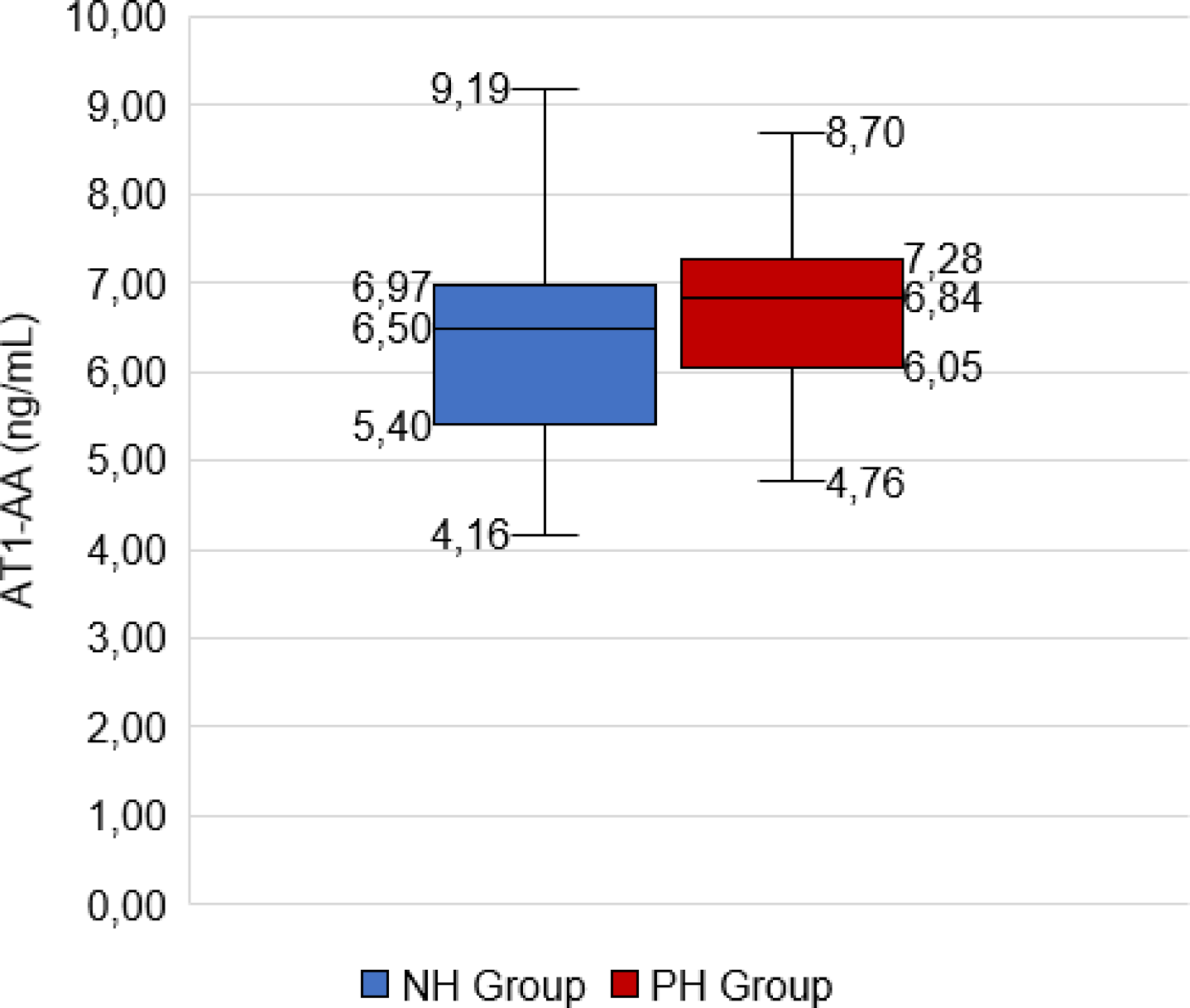
Circulating levels of angiotensin II Type 1 Receptor Autoantibodies (AT1-AA) a decade after the index pregnancy.

Circulating sFlt-1 levels were similar between groups, with a mean of 92 ± 34 pg/mL in the NH group and 90 ± 41 pg/mL in the PH group (*p* = 0.886) (Fig. 2). In the same way, AT1-AA levels showed no statistically significant difference between groups, with values of 6 ± 1 ng/mL in NH women and 7 ± 1 ng/mL in PH women (*p* = 0.060) (Fig. 3). In PH group, correlation analyses were performed between AT1-AA and LDLc, BMI, SBP and DBP. However, none significance was found *p* = 0.486; 0.636; 0.773 and 0.965, respectively.

Boxplot comparing circulating sFlt-1 levels in in women with prior PE (PH group, *n* = 103) and normotensive pregnancies (NH group, *n* = 102), approximately ten years after their index pregnancy. Statistical analysis was performed using Student’s t-test.

Boxplot comparing circulating AT1-AA levels in women with priorPE (PH group, *n* = 103) and normotensive pregnancies (NH group, *n* = 102), ten years after their index pregnancy. Statistical analysis was performed using Student’s t-test.

In the multivariate analysis, the pregnancy history effect was evaluated in the fully adjusted model, controlling for age, time since index pregnancy, sFlt-1, AT1-AA, HDL-C, BMI, and educational attainment. The variables time after index pregnancy, sFlt-1, AT1-AA, and HDL-C did not show a statistically significant effect in the initial multivariate model (*p* = 0.829, *p* = 0.697, *p* = 0.573, and *p* = 0.141, respectively). In the final model (Table 2), the same outcomes were assessed after adjustment for age, BMI, and education only. Participants in the PH group exhibited higher crude mean values than those in the NH group across all outcomes. The mean SBP was 133 in the PH group and 123 in the NH group; mean DBP was 89 and 81, respectively; and mean LDL-C was 107 and 90, respectively. Box’s M indicated heterogeneity of covariance matrices across groups (M = 19.511, F(6, 295326.213) = 3.199, *p* = 0.004). Levene’s test was non-significant for SBP (F(1, 202) = 3.560, *p* = 0.061) and LDL-C (F(1, 202) = 0.342, *p* = 0.560), but significant for DBP (F(1, 202) = 5.582, *p* = 0.019). Despite these assumption violations, the multivariate GLM showed a significant overall effect of pregnancy history (NH vs. PH group) on the combined outcomes set (Pillai’s Trace = 0.197, F(3, 70) = 5.720, *p* < 0.001. In univariate analyses, pregnancy history remained significantly associated with all three outcomes: SBP (F(1, 199) = 11.407, *p* = 0.001, partial η²=0.054), DBP (F(1, 199) = 20.410, *p* < 0.001, partial η²=0.093), and LDL-C (F(1, 199) = 8.673, *p* = 0.004, partial η²=0.042). Adjusted pairwise comparisons confirmed higher estimated marginal means in PH group. The adjusted mean differences were 7.559 mmHg for SBP (*p* = 0.001, 95% CI 3.145 to 11.972), 7.847 mmHg for DBP (*p* < 0.001), and 17.717 mg/dL for LDL (*p* = 0.004), all favoring PH group.

**Table 2:** Adjusted multivariate general linear model for systolic and diastolic blood pressures in women with a history of preeclampsia (PH group) compared to women with normotensive pregnancies (NH group), adjusted for age, body mass index, and educational attainment in the PERLA-Brazil cohort (*N* = 205).

Independent variable(s)	Dependent variable(s)	Analysis type	Test statistic	*p*-value	Effect size
PE history (NH vs. PH group); age; BMI; education	SBP, DPB, LDL-C	Multivariate GLM^a^	Pillai’s Trace Intercept: 0.237 Age: 0.107 BMI: 0.118 Education: 0.084	All < 0.001	Partial η² Intercept: 0.237 Age: 0.107 BMI: 0.118 Education: 0.084
PE history: PH group - NH group	SBP^b^	Adjusted pairwise comparison	Mean difference = 7.559	0.001^e^	95% CI [3.145–11.972]
	DBP^c^		Mean difference = 5.900	0.001^e^	95% CI [2.504–9.296]
	LDL-C^d^		Mean difference = 18.884	< 0.001^e^	95% CI [9.177–28.590]

NH: women with normotensive pregnancy history (n=102, 49.7%). PH: women with preeclampsia with severe features history (n=103, 50.3%). BMI: body mass index. SBP: systolic blood pressure. DBP: diastolic blood pressure. LDL-C: low-density lipoprotein cholesterol. *p-value < 0.05 indicates statistical significance. ^a^Design: Intercept + Age + BMI + educational attainment + PH/NH group. ^b^R²=0.261 (Adjusted R²=0.246). ^c^R²=0.270 (Adjusted R²=0.256). ^d^R²=0.102 (Adjusted R²=0.084). ^e^Adjustment for multiple comparisons: Bonferroni.

## Discussion

The present study investigated plasma levels of sFlt-1 and AT1-AA in women about ten years postpartum, comparing those with a history of PE (PH group) to women with prior normotensive pregnancies (NH group). We observed no significant differences in circulating sFlt-1 or AT1-AA levels between groups. The absence of significant differences in sFlt-1 and AT1-AA levels between groups at this extended follow-up contrasts with earlier postpartum study demonstrating persistent elevation of these biomarkers, suggesting that if elevation of these biomarkers occurs in the postpartum period following PE, such elevation may be transient^2^.

It is extensively recognized, that sFlt-1 and AT1-AA are as pivotal players in the pathophysiology of PE. AT1-AA, which act as agonists of the angiotensin II type 1 receptor, are known to promote vasoconstriction, hypertension, and endothelial dysfunction^5,12^. These autoantibodies also stimulate the production of sFlt-1, an antiangiogenic factor that sequesters VEGF and PlGF, leading to widespread maternal endothelial dysfunction and hypertension, which are hallmarks of the disease^7,16^. Indeed, the clinical utility of the sFlt-1/PlGF ratio as a diagnostic tool underscores the relevance of sFlt-1 in identifying and monitoring the disease^8^. Furthermore, sFlt-1 has been implicated in promoting oxidative stress and apoptosis in preeclamptic trophoblasts, reinforcing its mechanistic role during the active phase of PE^9^.Existing literature also supports that exposure to AT1-AA and higher levels of sFlt-1 during pregnancy can induce long-term changes in cardio-vascular structure and function^6,11,17^. Some preclinical and clinical studies have indicated that these factors might remain elevated for several months postpartum in a subset of women with a history of PE, potentially contributing to persistent endothelial dysfunction and increased CVD risk. For example, Hubel et al. (2007) detected AT1-AA in women 18 months postpartum^2^, suggesting that biomarker elevation may persist beyond the immediate postpartum period. These observations provided the rationale for our investigation into their very long-term persistence (approximately ten years after pregnancy).

However, the absence of significant differences in our cohort at this extended follow-up may reflect biological resolution or compensatory mechanisms occurring before a decade postpartum in most women with prior PE. Furthermore, the altered levels of these biomarkers after pregnancy could be limited to a smaller, highly specific subset of women not captured within our cohort – potentially due to limited sample size.

The clearest and most consistent between-group differences were observed in cardiovascular risk indicators, particularly blood pressure and LDL-C. Accordingly, while sFlt-1 and AT1-AA were the primary biological targets of this investigation, the strongest empirical signal emerged from the cardiovascular outcomes. This divergence suggests that the long-term sequelae of PE may be captured more effectively by downstream vascular and metabolic alterations than by persistent circulating levels of the original biomarkers. In this cohort, the absence of significant differences in sFlt-1 and AT1-AA after one decade does not negate the enduring clinical impact of PE; rather, it indicates that the chronic risk associated with this condition may be mediated through broader cardiovascular remodeling. These findings are consistent with the concept that PE may function as a vascular stress test, unmasking or amplifying an underlying predisposition to adverse cardiovascular profiles that remains evident years after delivery^18,19^. Importantly, the cardiovascular phenotype observed here was not uniform across all domains. The strongest and most consistent associations were blood pressure and LDL-C, witches reinforces that the biological footprint of PE may persist primarily through hemodynamic and metabolic pathways rather than the increased sFlt-1 and AT1-AA plasma levels. The observed differences (particularly the nearly 17 mg/dL gap in LDL-C and the significant elevation in blood pressure) are clinically meaningful, as they represent a heightened trajectory toward chronic hypertension and atherosclerotic CVD.

From a clinical perspective, these results underscore the importance of long-term cardiovascular surveillance in women with PE history especially in low- and middle-income countries such as Brazil, where care is often provided during pregnancy but insufficiently sustained thereafter within health systems and policies.

## Conclusions

Despite the absence of differences of sFlt-1 and AT1-AA plasma levels, ten years after PE, our study clearly corroborates the widely recognized long-term cardiovascular sequelae associated with a history of PE. Women in the PH group exhibited significantly higher SBP and DBP, a less favorable BMI profile along with a markedly higher prevalence of hypertension requiring medication and elevated LDL-C levels compared to the NH group. These findings reassert that a history of PE is associated with increased cardiovascular risk throughout life.

The findings of this study also demonstrate that a history of PE serves as an independent risk factor for elevated blood pressure and dyslipidemia in the postpartum period. Even after controlling for potential confounders such as age, BMI, and educational attainment, women who experienced PE maintained significantly higher levels of SBP, DBP, and LDL-C.

These results support the “vascular stress test” hypothesis, suggesting that PE unmasks or exacerbates a pre-existing cardiovascular vulnerability that persists long after delivery.

The significant contribution of BMI and age to blood pressure levels highlight the necessity for long-term longitudinal follow-up and early lifestyle or pharmacological interventions for women with a history of PE to mitigate their enduring risk of cardiovascular events.

### Limitations

The cross-sectional design with a single biomarker measurement at approximately 10 years postpartum precludes inference about biomarker trajectories. The absence of intermediate measurements (e.g., at 1, 5, and 10 years postpartum) prevents characterization of temporal dynamics in biomarker changes.

The study had limited powered to detect differences in sFlt-1 and AT1-AA between the groups. Since the original sample size was calculated based on the variance of LDL-C levels (the primary endpoint of the initial study design) the current analysis may have lacked the statistical sensitivity required to identify subtle variations in these specific biomarkers. Consequently, the observed low post-hoc power increases the risk of Type II errors, and the non-significant findings should be interpreted with caution as they may reflect an underpowered cohort rather than a true absence of biological effect.

Recruitment methodology introduces additional constraints. Social media and radio recruitment (Phase 3) yielded 92% of the final sample, whereas systematic hospital-based recruitment (Phase 2) achieved only 2% participation (6/226 eligible women). This recruitment pattern suggests that the cohort comprises women with higher levels of health consciousness, media exposure, and research engagement, who may differ systematically from women with prior PE who do not self-refer. Consequently, findings regarding biomarkers and cardiovascular risk may not generalize to all women with PE history, particularly those with lower health literacy, limited media exposure, or more severe postpartum complications.

Finally, the retrospective design and reliance on self-reported clinical and demographic data introduce potential recall bias, while the regionally specific nature of the cohort limits external validity.

### Future perspectives

To address the selection bias identified in this study, future research should employ systematic, population-based recruitment strategies rather than relying on self-referral. This includes identifying all women with a history of PE in defined geographic regions during specific periods, implementing active outreach with multiple contact attempts, and offering adequate compensation and flexible participation options to maximize response rates. Comparison of characteristics between participants and non-participants would help quantify selection bias and assess cohort representativeness.

Future research should prioritize prospective, longitudinal studies initiated in pregnancy, incorporating serial biomarker measurements and comprehensive objective data collection. A critical direction is the identification of novel, persistent biological or physiological markers beyond sFlt-1 and AT1-AA that directly correlate with long-term cardiovascular risk, ideally through multi-omics approaches in larger, diverse cohorts. Mechanistic studies examining vascular, metabolic, and structural adaptations underlying persistent cardiovascular vulnerability are also warranted. Our findings reinforce that, despite sFlt-1 and ATT1-AA normalization, PE confers enduring CVD risk. This underscores the need for comprehensive, long-term postpartum follow-up, targeted prevention strategies, and the development of advanced tools for refined risk assessment, targeted intervention, and reliable prediction models in clinical practice and healthcare policy.

## Supplementary Information

Below is the link to the electronic supplementary material.

Supplementary Material 1

## Data Availability

The datasets used and/or analysed during the current study are available from the corresponding author on reasonable request.

## Abbreviations

AT1-AA: Autoantibodies against the angiotensin II type 1 receptor
ACOG: American College of Obstetricians and Gynecologists
AT1: Angiotensin II type 1 receptor
BAL200: Bioimpedance scale model BAL200 (Cadence Smart Care^®^)
BMI: Body mass index
CAAE: Certificate of Presentation for Ethical Consideration (Brazilian ethics approval registry)
CVD: Cardiovascular disease
DBP: Diastolic blood pressure
DM: Diabetes mellitus
ELISA: Enzyme-linked immunosorbent assay
FAFAR: Faculty of Pharmacy
HDL: High-density lipoprotein
IBM: International business machines
IQR: Interquartile range
LDL: Low-density lipoprotein
NH: Normotensive pregnancy history group
PE: Preeclampsia
PERLA: Preeclampsia and Latent Cardiovascular Risk After Delivery
PlGF: Placental growth factor
SBP: Systolic blood pressure
SD: Standard deviation
sFlt-1: Soluble fms-like tyrosine kinase-1
SPSS: Statistical package for the social sciences
UFMG: Federal University of Minas Gerais
VEGF: Vascular endothelial growth factor
WHO: World Health Organization
