# Supplementary material for: Circulating levels of sFlt-1 and AT1-AA in women with prior preeclampsia: a 10-year postpartum cross-sectional analysis (PERLA-Brazil study)
Source: Sci Rep. 2026 May 6;16:20845. doi: 10.1038/s41598-026-50409-1 (PMC13338439; doi:10.1038/s41598-026-50409-1)
Supplement: Supplementary file 1 — Supplementary Material 1 [file 41598_2026_50409_MOESM1_ESM.pdf]

Change of authorship request form - Journals (pre-acceptance)

Important information. Please read.

- This form should be used by authors to request any change in authorship (adding/deleting authors) including changes in corresponding authors. This form should not be used for name changes. Please fully complete all sections. Use black ink and block capitals and provide each author's full name with the given name first followed by the family name.

- By signing this declaration, all authors guarantee that the order of the authors are in accordance with their scientific contribution, if applicable as different conventions apply per discipline, and that only authors have been added who made a meaningful contribution to the work.

- Please note, in author collaborations where there is formal agreement for representing the collaboration, it is sufficient for the representative or legal guarantor (usually the corresponding author) to complete and sign the Authorship Change Form on behalf of all authors, **next to the added/removed author(s)**. **(Complete Section 3, followed by Section 6.)**
  In author collaborations where there is no formal agreement for representing the collaboration and **there are more than 10 authors**, one may sign for all, provided the signer appends correspondence that attests that each of the authors have agreed to the change **and the added/removed authors sign the form**. **(Complete Section 3, followed by Section 6.)**

- Please note, we cannot investigate or mediate any authorship disputes. If you are unable to obtain agreement from all authors (including those who you wish to be removed) you must refer the matter to your institution(s) for investigation. Please inform us if you need to do this.

- If you are not able to return a fully completed form within **30 days** of the date that it was sent to the author requesting the change, we may have to withdraw your manuscript. We cannot publish manuscripts where authorship has not been agreed by all authors (including those who have been removed).

- Incomplete forms will be rejected.

- Please return/upload this form, fully completed, to the Journals Editorial Office. The Journal and/or Publisher will consider the information you have provided to decide whether to approve the proposed change in authorship. We may decide to contact your institution for more information or undertake a further investigation, if appropriate, before making a final decision.

SPRINGER NATURE

Change of authorship request form - Journals (pre-acceptance)

Section 1: Please provide the current title of manuscript

Manuscript ID no.: 7017534e-244e-466b-a936-c78efcd36027

Title: Circulating Levels of sFlt-1 and AT1-AA in Women with Prior Preeclampsia: A 10-Year Postpartum Cross-Sectional Analysis (PERLA-Brazil Study)

Section 2: Please provide the previous authorship, in the order shown on the manuscript before the changes were introduced. Please indicate the corresponding author by adding (CA) behind the name.

	First name(s)	Family name	ORCID or SCOPUS id, if available
1st author	Thaíse Emilia	Moreira da Silva	https://orcid.org/0000-0002-6378-2818
2nd author	Isabella	Macedo Costa	https://orcid.org/0000-0002-9919-1358
3rd author	Ana Paula	Silva Ferreira	https://orcid.org/0009-0003-7281-5698
4th author	Thays	Santos da Costa	https://orcid.org/0009-0000-7035-3088
5th author	Luiza	Oliveira Perucci	https://orcid.org/0000-0003-1736-2721
6th author	Luci	Maria Sant'Ana Dusse	https://orcid.org/0000-0002-7484-1684
7th author	Jussara	Mayrink	https://orcid.org/ 0000-0001-5957-4365
8th author	Patrícia	Nessralla Alpoim	https://orcid.org/0000-0002-3602-2645
9th author			
10th author			

Please use an additional sheet if there are more than 10 authors.

SPRINGER NATURE Springer Nature is one of the world’s leading global research, educational and professional publishers, created in May 2015 through the combination of Nature Publishing Group, Palgrave Macmillan, Macmillan Education and Springer Science+Business Media.

Change of authorship request form - Journals (pre-acceptance)

SPRINGER NATURE

Section 3: Please provide a justification for change. Please use this section to explain your reasons for changing the authorship of your manuscript, e.g. what necessitated the change in authorship? Please refer to the (journal) policy pages for more information about authorship. Please explain why omitted authors were not originally included and/or why authors were removed on the submitted manuscript.

Juliano Armstrong Arnosti, who holds a degree in Statistics, was invited to participate in this study to support the assessment of the correlations between the biomarkers and the other parameters evaluated in the women included in the analysis. His expertise was essential to the development of the Multivariate General Linear Model, with systolic blood pressure, diastolic blood pressure, and low-density lipoprotein cholesterol entered as dependent variables and pregnancy history (normotensive pregnancy history group vs. preeclampsia with severe features history group) as the fixed between-subjects factor. His contribution was fundamental to ensuring that the statistical analyses were carried out as comprehensively as possible, thereby adding important insights to the article's results.

Section 4: Proposed new authorship. Please provide your new authorship list in the order you would like it to appear on the manuscript. Please indicate the corresponding author by adding (CA) behind the name. <u>If the Corresponding Author has changed, please indicate the reason under section 3</u>.

	First name(s)	Family name (this name will appear in full on the final publication and will be searchable in various abstract and indexing databases)	Affiliated institute	E-mail address
1st author	Thaíse Emilia	Moreira da Silva	Department of Clinical and Toxicological Analysis, Faculty of Pharmacy Federal University of Minas Gerais, Belo Horizonte, Brazil	thaiseemiliams@gmail.com
2nd author	Isabella	Macedo Costa	Department of Clinical and Toxicological Analysis, Faculty of Pharmacy Federal University of Minas Gerais, Belo Horizonte, Brazil	mcostaisabella@gmail.com
3rd author	Ana Paula	Silva Ferreira	Department of Clinical and Toxicological Analysis, Faculty of Pharmacy, Federal University of Minas Gerais, Belo Horizonte, Brazil	anapsilvaferr@gmail.com
4th author	Thays	Santos da Costa	Department of Clinical and Toxicological Analysis, Faculty of Pharmacy, Federal University of Minas Gerais, Belo Horizonte, Brazil	thaysantosc23@gmail.com
5th author	Luiza	Oliveira Perucci	University of California, San Diego, La Jolla, California, United States	luizaperucci@gmail.com
6th author	Juliano	Armstrong Arnosti	Federal University of Paraná, Curitiba, Brazil	juarmstrong@gmail.com
7th author	Luci	Maria Sant'Ana Dusse	Department of Clinical and Toxicological Analysis, Faculty of Pharmacy, Federal University of Minas Gerais, Belo Horizonte, Brazil	lucidusse@gmail.com
8th author	Jussara	Mayrink	Faculty of Medicine, Federal University of Minas Gerais, Belo Horizonte, Brazil	jussaramayrink@gmail.com
9th author	Patrícia	Nessralla Alpoim	Department of Clinical and Toxicological Analysis, Faculty of Pharmacy, Federal University of Minas Gerais, Belo Horizonte, Brazil	patnessralla@yahoo.com.br
10th author				

Please use an additional sheet if there are more than 10 authors.

SPRINGER NATURE Springer Nature is one of the world's leading global research, educational and professional publishers, created in May 2015 through the combination of Nature Publishing Group, Palgrave Macmillan, Macmillan Education and Springer Science+Business Media.

SPRINGER NATURE

Change of authorship request form - Journals (pre-acceptance)

Section 5: Author contribution, Acknowledgement and Disclosures. Please use this section to provide a new disclosure statement and, if appropriate, acknowledge any contributors who have been removed as authors and ensure you state what contribution any new authors made (if applicable per the journal or book (series) policy). Please ensure these are updated in your manuscript - after approval of the change(s) - as our production department will not transfer the information in this form to your manuscript.

New acknowledgements:

The authors express their profound gratitude to all the women who generously participated in this clinical study. We also acknowledge with deep appreciation the financial support provided by FAPEMIG (APQ-00719-18 and APQ-02600-18) and CNPq/Brazil (Grant Number 408985/2021) and the CAPES.

New Disclosures (financial and non-financial interests, funding):

This work was supported by the Fundação de Amparo à Pesquisa do Estado de Minas Gerais (FAPEMIG) (APQ-00719-18 and APQ-02600-18) and Conselho Nacional de Desenvolvimento Científico e Tecnológico (CNPq/Brazil) (Grant Number 408985/2021).and the Coordenação de Aperfeiçoamento de Pessoal de Nível Superior (CAPES).

New Author Contributions statement (if applicable per the journal policy):

Juliano Armstrong Arnosti (https://orcid.org/ 0000-0002-3685-5645):
I declare that I participated in the reviewing statistical analysis and I have seen and approved the final version. I have no conflicts of interest.

State 'Not applicable' if there are no new authors.

SPRINGER NATURE Springer Nature is one of the world's leading global research, educational and professional publishers, created in May 2015 through the combination of Nature Publishing Group, Palgrave Macmillan, Macmillan Education and Springer Science+Business Media.

SPRINGER NATURE

Change of authorship request form - Journals (pre-acceptance)

Section 6: Declaration of agreement. *All* authors, unchanged, new and removed *must* sign this declaration.
(NB: Please print the form, (docu)-sign and return/upload a scanned copy. Please note that signatures that have been inserted as an image file are acceptable as long as it is handwritten. Typed names in the signature box are unacceptable.) *** Please delete as appropriate. Delete all of the bold if you were on the original authorship list and are remaining as an author.**

	First name	Family name		Signature	Date
1st author	Thaíse Emilia	Moreira da Silva	I agree to the proposed new authorship shown in section 4 **/and the addition/removal*of my name to the authorship list** /and the proposed change in corresponding author	[illegible]	April 17, 2026
2nd author	Isabella	Macedo Costa	I agree to the proposed new authorship shown in section 4 **/and the addition/removal*of my name to the authorship list** /and the proposed change in corresponding author	Isabella Macedo Costa	April 17, 2026
3rd author	Ana Paula	Silva Ferreira	I agree to the proposed new authorship shown in section 4 **/and the addition/removal*of my name to the authorship list** /and the proposed change in corresponding author	Ana Paula Silva Ferreira	April 17, 2026
4th authors	Thays	Santos da Costa	I agree to the proposed new authorship shown in section 4 **/and the addition/removal*of my name to the authorship list** /and the proposed change in corresponding author	Thays Santos da Costa	April 17, 2026
5th author	Luiza	Oliveira Perucci	I agree to the proposed new authorship shown in section 4 **/and the addition/removal*of my name to the authorship list** /and the proposed change in corresponding author	Luiza Perucci	April 17, 2026
6th author	Juliano	Armstrong Arnosti	I agree to the proposed new authorship shown in section 4 **/and the addition/removal*of my name to the authorship list** /and the proposed change in corresponding author	[illegible]	April 17, 2026
7th author	Luci	Maria Sant'Ana Dusse	I agree to the proposed new authorship shown in section 4 **/and the addition/removal*of my name to the authorship list** /and the proposed change in corresponding author	Luci Dusse	April 17, 2026

SPRINGER NATURE Springer Nature is one of the world's leading global research, educational and professional publishers, created in May 2015 through the combination of Nature Publishing Group, Palgrave Macmillan, Macmillan Education and Springer Science+Business Media.

Change of authorship request form - Journals (pre-acceptance)

	First name	Family name		Signature	Date
8th author	Jussara	Mayrink	I agree to the proposed new authorship shown in section 4 /**and the addition/removal*of my name to the authorship list** /and the proposed change in corresponding author	Jussara Mayrink	April 17, 2026
9th author	Patrícia	Nessralla Alpoim	I agree to the proposed new authorship shown in section 4 /**and the addition/removal*of my name to the authorship list** /and the proposed change in corresponding author	Patrícia Nessralla Alpoim	April 17, 2026
10th author			I agree to the proposed new authorship shown in section 4 /**and the addition/removal*of my name to the authorship list** /and the proposed change in corresponding author		

Please use an additional sheet if there are more than 10 authors.

In case of author collaborations with formal agreement:

	Name of consortium/consortia	First name	Family name		Signature	Date
Representative/legal guarantor				I agree to the proposed new authorship shown in section 4 /**and the addition/removal*of my name to the authorship list** /and the proposed change in corresponding author		

Both added/removed authors should complete the information in the first table under Section 6.

---- End of form ----

SPRINGER NATURE Springer Nature is one of the world's leading global research, educational and professional publishers, created in May 2015 through the combination of Nature Publishing Group, Palgrave Macmillan, Macmillan Education and Springer Science+Business Media.
